# Effectiveness of a web-based education program to improve vaccine storage conditions in primary care (Keep Cool): study protocol for a randomized controlled trial

**DOI:** 10.1186/s13063-015-0824-9

**Published:** 2015-07-14

**Authors:** Anika Thielmann, Anja Viehmann, Birgitta M. Weltermann

**Affiliations:** Institute for General Medicine, University Hospital Essen, University of Duisburg-Essen, Hufelandstr. 55, 45147 Essen, Germany

**Keywords:** Vaccine management, Primary care, Vaccine cold chain, Education, E-learning, Quality improvement

## Abstract

**Background:**

Immunization programs are among the most effective public health strategies worldwide. Adequate vaccine storage is a prerequisite to assure the vaccines’ effectiveness and safety. In a questionnaire survey among a random sample of German primary care physicians, we discovered vaccine storage deficits: 16 % of physicians had experience with cold chain breaches either as an error or near error, 49 % did not keep a temperature log, and 21 % did not use a separate refrigerator for vaccine storage. In a recent feasibility study of 21 practice refrigerators, we showed that these were outside the target range 10.2 % of the total time with some single refrigerators being outside the target range as much as 66.3 % of the time. These cooling-chain deficits are consistent with the international medical literature, yet an effective, easy to disseminate, practice-centered intervention to improve storage conditions is lacking.

**Methods/design:**

This randomized intervention trial will be conducted in a random sample of primary care practices. Based on continuous temperature recordings over 7 days, all practices with readings outside the target range for vaccine storage (+2 °C to +8 °C) will be randomly allocated to a web-based education program or a waiting list control group. The practice physicians and their teams constitute the target population. Participants will be educated about best practices in vaccine storage and will receive a manual including storage checklists and templates for temperature documentation. In all practices, temperatures of the vaccine refrigerators will be monitored continuously using a data logger with a glycol probe as a surrogate for vaccine vial temperature. The effectiveness of the web-based education program will be determined after 6 months in terms of the proportion of refrigerators with vaccine vial temperatures within the target range (+2 °C to +8 °C) during 7-day temperature logging. Secondary outcome parameters include temperature monitoring, no critically low temperatures (≤ -0.5 °C), compliance with storage recommendations, knowledge of good vaccine storage conditions, and assignment of personnel as vaccine storage manager and backup.

**Discussion:**

Keep Cool will develop and evaluate a web-based education program to improve vaccine storage conditions in primary care and thereby ensure immunization safety and effectiveness.

**Trial registration:**

DRKS00006561 (date of registration: 20 February 2015)

## Background

Immunizations are among the most effective and cost-effective public health strategies worldwide [1]. Adequate vaccine storage is a prerequisite to maintain effectiveness and safety of the vaccines. The maintenance of the vaccine cold chain (2 °C to 8 °C) is of utmost importance to assure vaccine potency [2]. Detailed analyses of vaccine temperature sensitivities documented freezing of vaccines as being more critical than heating, at least in our moderate Western European climate. Modern adsorbed hepatitis B vaccines were documented to be the most sensitive vaccines with a freezing point of -0.5 °C [2]. At this temperature, irreversible precipitates of aluminum-containing adsorbents begin to form, which decrease the potency and can induce local irritation upon injection [2, 3].

In a cross-sectional web-based questionnaire survey, we analyzed vaccination management among a 10 % random sample of primary care physicians from one of Germany’s largest federal states, North Rhine-Westphalia [4] using international recommendations for vaccine storage [5–9]. The survey (n = 211) revealed that 16 % of the primary care physicians have experience with cold chain breaches either as an error or near error; 8 % do not regularly control their storage with regard to vaccine wrapping, vaccine expiration date, and refrigerator temperature; 49 % do not keep a storage temperature log; and 21 % fail to use a separate refrigerator solely for vaccine storage. In a recent feasibility study, we observed that the correct temperature range of 2 to 8 °C was not consistently maintained in seven of 21 (33.3 %) vaccine refrigerators from teaching practices. Refrigerators were outside the target range 10.2 % of the total time, with single refrigerators outside the target range for as much as 66.3 % of the time. These results are in line with studies worldwide, which discuss the following key problems related to vaccine storage: 1) temperatures outside the target range [10, 11], 2) lack of adequate temperature measurement devices [12–14], 3) lack of continuous temperature documentation [4, 12–14], 4) use of inadequate refrigerators [9, 10, 12], 5) lack of separate refrigerators [13–15], 6) inadequate storage practices [2, 12, 14, 15], 7) lack of designated personnel [14], and 8) insufficient staff training and guidance [12, 14, 16–18].

Prior studies addressing these deficits used the following intervention components either alone or in combination: 1) written educational materials, 2) introduction of thermometers with or without feedback on temperature readings by either graphic display or telephone advice to personnel, and 3) 1:1 onsite education with inspection of refrigerators. In 2002, a prospective intervention study of 721 US primary care practices achieved improved compliance with several storage recommendations of up to 19 % after 3 months following the distribution of a manual, a thermometer and a feedback checklist [12]. Similar results with improvements ranging from 13 to 23 % after 4 weeks were shown in a Korean study of 39 private clinics, which provided onsite 1:1 education including the distribution of a manual. This study also documented improved awareness of various vaccine storage criteria ranging from 3 % to 29 % [16]. With regard to reaching the targeted temperature range, the best result was observed in an Australian study of 50 primary care practices: onsite education and the distribution of min/max-thermometers led to a fourfold increase of practices with refrigerator temperatures in the target range [18]. The two studies available, which provided long-term follow-ups after 6 months, 1 year, and 5 years, documented a tendency for improved temperatures in the populations studied but also reported fluctuations over time in 25 % of the practices with initially optimal temperatures [19, 20]. The repetitive documentation of such fluctuations in intervention as well as control groups underlines the need for structural change as part of educational interventions, including the long-term implementation of practice routines for daily temperature monitoring.

Following recommendations on effectively changing medical practice [21], our study will use a complex approach focusing on the individual as well as the organizational level by encouraging structural changes. The intervention will address both professional groups involved in the management of vaccines, that is, the physician managers in charge, as well as the practice assistants who handle vaccines. Based on didactic approaches such as information tailoring and confidence-based learning, relevant information will be presented in a web-based education program, which tailors information to each users’ knowledge. Prospectively, this e-learning approach will allow for widespread dissemination at low cost.

Keep Cool aims to evaluate the effectiveness of a web-based education program to improve vaccine storage conditions in primary care practices. The main outcome used to determine the effectiveness of the intervention is the proportion of refrigerators with vaccine vials within the target range (+2 °C to +8 °C) during 7-day monitoring after 6 months.

## Methods/design

### Study design

**Fig. 1:**
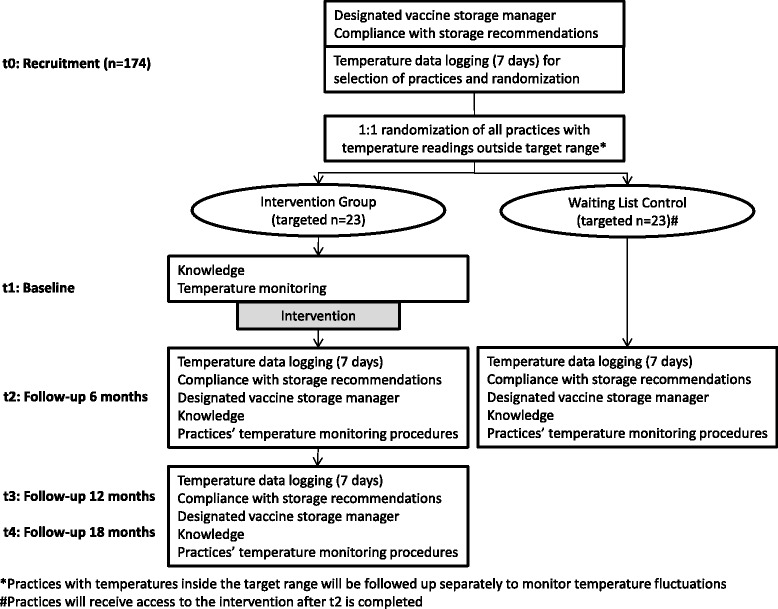
Study overview

Keep Cool is a prospective, randomized, controlled intervention trial, which will be performed in a random sample of primary care practices (see Fig. 1). After telephone recruitment, all practices will be visited by a study assistant to set up temperature data loggers (t0). Based on continuous temperature recordings over 7 days, all practices with readings outside the target range for vaccine storage (+2 °C to +8 °C) will be randomly allocated (1:1) to an intervention group or a waiting list control group. Practices with temperatures within the target range will be followed separately to monitor temperature fluctuations. Baseline data on knowledge will be collected at t1 prior to the start of the intervention, whereas the compliance with storage recommendations and the assignment of personnel as vaccine storage manager and backup will have already been documented by the study assistant during recruitment (t0). After baseline measurements at t1, the intervention group will receive access to the e-learning program. Follow-up data will be obtained after 6 months (t2) and, to study the intervention’s long-term effectiveness, after 1 year (t3) and 18 months (t4). After the intervention phase is completed, the waiting list control group will receive access to the e-learning program.

Ethical approval was obtained from the Ethic Commission of the Medical Faculty of the University Hospital Essen, University of Duisburg-Essen (reference number: 14-6118-BO, date of approval: 04/02/2015). Keep Cool follows the Ethical Principles of the World Medical Association Declaration of Helsinki (WMA 1964).

### Study population

The target population of this randomized controlled trial is primary care physicians and their practice personnel. The majority of these personnel completed a 3-year vocational training with a degree (practice assistants). A few practices also employ secretaries, nurses or personnel without a degree, but with on-the-job training. The intervention will address all staff members handling vaccines regardless of their prior training. Practices are eligible if they administer vaccinations and are situated within a 50-km radius of Essen, Germany. All participating physicians and practice personnel will sign an informed consent form.

### Intervention: web-based education program

Physicians and practice personnel will participate in a web-based education program on the storage of vaccines according to international best practices (for example, WHO and CDC). To ensure the target populations’ acceptance of the program, we will ask primary care physicians and practice assistants of academic teaching practices of our research network to join recurrent focus group sessions. This group will facilitate the development, adaptation, implementation, and evaluation phase of the intervention.

The e-learning program will be developed using a teaching software that allows the tailoring of information to each user group (that is, physicians and medical assistants) and each individual’s previous response behavior. Following confidence-based learning, the e-learning program will require participants to a) answer concrete vaccine storage-related questions and b) indicate the degree to which they feel confident in the correctness of each answer. Depending on the respondents’ answers, that is, correctness and confidence of answers, each participant will receive learning input with subsequent learning-success-adjusted repetitions of questions to achieve optimal memorizing effects. All participants will receive tailored feedback during the course of answering questions on an item level and for all items after completion of the program.

Five educational topics will be addressed by the web-based education program:

1. Requirements for technical equipment including refrigerator criteria; types of thermometers; equipment maintenance.
2. Vaccine temperature sensitivity, target temperature, and daily reviewing and recording of temperatures: freezing thresholds of freeze-sensitive vaccines; refrigerator target range (2-8 °C = 35.6-46.4 °F); necessity of daily recording; recording criteria; need for long-term temperature logs; and use of data logs (paper-based or electronically).
3. Principles of vaccine storage inside a refrigerator including the preparation of refrigerators for vaccine storage, background knowledge about the use of domestic refrigerators for vaccine storage (incl. refrigerator cycles, temperature zones), necessity of a separate refrigerator for the sole purpose of vaccine storage, standards for vaccine storage within refrigerators, and use of storage aids.
4. Vaccine cold chain-related processes including vaccine arrival procedures, periodic manual storage control, and procedures for dealing with cold chain breaches and equipment failure.
5. Responsibilities of practice personnel including the assignment of personnel as vaccine storage manager and backup.

To facilitate long-term changes at the organizational level, all practices will be advised to assure the following two structural components: 1) the assignment of personnel as vaccine storage manager and at least one backup and 2) the set-up of a comprehensive temperature monitoring system. This includes reviewing temperatures using a standard thermometer, recording temperatures in a temperature log, taking immediate action if needed, and retaining temperature logs. In addition to a storage manual summarizing the program content, the following material will be made available as a web download: a “temperature log template,” guides for “preparing a refrigerator for vaccine storage,” “storing vaccines in the refrigerator,” “refrigerator maintenance,” plans for “routine vaccine management,” and “emergency response,” as well as a summarizing overview of the “tasks of the vaccine coordinator.”

### Outcomes

*Primary outcome:* The proportion of refrigerators with vaccine vials within the target range (+2 °C to +8 °C) during a 7-day monitoring at 5-minute intervals after 6 months. Temperature inside the target range is chosen as the primary outcome, because it is of utmost importance that nonfreezing vaccines are stored in this temperature range. When absorbed mono- and combination vaccines reach their individual freezing thresholds, their chemical structures irreversibly change. This can lead to increased local reactions and reduced effectiveness. The most sensitive vaccines are hepatitis B vaccines with a freezing threshold of -0.5 °C [2]. A thermal analysis of frequently used types of refrigerators by the US Department of Commerce [22, 23] showed that vaccine vial temperatures vary depending on the refrigerator type, loading density and location of vaccines inside the refrigerator. Typical refrigerators used in German primary care are the types “freezerless” (single refrigerator compartment without a freezer) and “dual zone” (separate compartments for refrigerator and freezer, both with individual doors), both so-called domestic refrigerators. Based on different scenarios, that is, door openings and loading density, temperatures can vary by +/-2.3 °C in the type “freezerless” and by +/-4.5 °C in the type “dual zone” [22, 23]. The maintenance of the temperature range of +2 to +8 °C, as recommended by vaccine manufacturers and public health authorities worldwide, is hence justified and therefore serves as a primary outcome measure.

Temperatures will be measured via a data logger that is approved by the US Center for Disease Control (CDC), and the same type will be distributed to each practice in order to ensure consistency of temperature readings across locations. Continuous measurements over 7 days are planned for baseline (t0) and continuously thereafter, with follow-ups after 6 months (t2), 1 year (t3) and 18 months (t4). Continuous measurements are preferable to single-point measurements because many practices use nonpurpose built refrigerators (so-called domestic refrigerators). Such refrigerators are known to have temperature cycles that result from their on-/off-construction: using the physical principle of the Carnot cycle, they cool down to a specific temperature, after which the cooling department is shut off until a higher temperature is reached [22, 23]. To analyze such temperature fluctuations, we chose a logging interval of one reading every 5 minutes. Similar logging rates were used in prior studies [12, 14, 17, 19, 20]. The logging period of 7 days ensures that at least one regular working week and one weekend are included. This takes differences in patient flow and refrigerator door openings into account (for example, typically more patients on Mondays).

*Secondary outcomes* follow international best practices and guidelines:

1. Temperature monitoring - Improvement in temperature monitoring with optimal monitoring defined as electronic or paper-based recording with a minimum of two readings per day for the previous 5.5 months (5 % missing entries allowed). Prior studies indicated that temperature fluctuations without adequate adjustments over time are a main barrier to intervention success. To overcome this barrier, this intervention program will emphasize temperature monitoring, including temperature reviewing, recording, taking immediate action if needed and retention of temperature logs.
2. Refrigerators not freezing - The proportion of practices with vaccine vials not reaching critically low temperatures (-0.5 °C or lower) will be determined.
3. Compliance with storage recommendations - Improvement in actual refrigerator-related storage quality will be assessed at practice level according to the following examples:

- Refrigerator is used exclusively for vaccine storage.
- Standard thermometer is located in refrigerator.
- No vaccines are stored in refrigerator door.
- Vaccines are stored in their original wrapping.
- e Vaccines are stored in the center of the refrigerator.
- Use of a stock management system (for example, “earliest expiry first out (EEFO)” principle) and regular manual stock control.

4. Knowledge about vaccine storage - Improvement in knowledge of good vaccine storage conditions in physicians and practice personnel will be studied using a standardized questionnaire.
5. Assignment of personnel - During the initial practice visit the study assistant will ask if practices currently have assigned personnel as vaccine storage manager and backup. This is considered an important structural characteristic to maintain long-term changes at practice level.

### Measurement instruments

#### Temperature data logging

An important distinguishing factor from previous studies, but in line with international best practices [5–7], is the use of a data logger with one high-accuracy thermistor probe immersed in glycol. In contrast to a standard air probe, the slow-reacting glycol probe resembles the temperature changes of the vaccine vials, whereas the fast-reacting air probe only shows the ambient air temperatures inside the refrigerator and the effects of door openings on refrigerator temperatures.

Specifications and set-up of the chosen data logger are described below:

1. Type - EL-GFX-DTP.
2. Manufacturer - Lascar Electronics Ltd, UK.
3. Probe - High-accuracy thermistor probe immersed in glycol.
4. Accuracy for probe within the operating range -5 °C to +10 °C - +/− 0.1 °C.
5. Logging rate - 1 reading per 5 minutes.

According to standards, this data logger will be placed in the center of the refrigerator for continuous recording. In practices using more than one refrigerator for vaccine storage, the refrigerator most frequently used and closest to the reception desk will be selected.

#### Self-administered questionnaire (knowledge and temperature monitoring)

The questionnaire will consist of a knowledge part (part 1) and a part addressing current structures and procedures used for monitoring of temperatures (part 2). The knowledge part will be developed drawing on the international body of recognized best practices and guidelines (for example, WHO), official checklists used in other countries (UK, US, and AUS) and previous studies (for example, [12, 14, 16, 17, 20]). Items will correspond to the five educational topics detailed above. In addition, sociodemographic characteristics of physician and practice personnel (sex, age, years in practice/job, part-/full-time, and job content) and practice characteristics (single/group, number of physicians/medical assistants in practice, number of patients quarterly, average number of vaccinations daily, age structure of patients, and urban/rural) will be included. In part two, the current structures and procedures used for the monitoring of temperatures will be obtained. Participants will complete part 1 and 2 of the questionnaire after accessing the web-based program, but before starting with the educational program itself (t1). Follow-up measurements will use a paper-based version of part 1 of the questionnaire, handed out and collected when downloading follow-up data logger readings (t2 and t4). Inspections of temperature logbooks at t2 and t4 will serve as the follow-up measurement for part 2.

#### Storage checklist

Changes in the compliance with storage recommendations will be assessed using a checklist completed by the study assistant. This checklist will be a modification of a tool used in the previously mentioned intervention study by Lee et al. (2012) [16] and draws on the same sources listed above. This checklist is completed when the data loggers are set up (t0) and again when the temperature readings are downloaded (t2, t3, t4).

##### Waiting list control group

The control group will consist of practices that have temperature readings outside the target range during baseline data collection. The control group will be monitored via a temperature data logger but will receive access to the e-learning program only after t2. By including a control group, an intervention bias due to the set-up of data loggers is taken into account. The control group is closest to regular everyday care and serves as a baseline to better determine the effectiveness of the intervention.

### Safety issues

All practices will be informed in detail about their vaccine refrigerator temperature. The tailored education and the download material will address the issue of how to deal with temperature breaches. Practices with temperature readings outside the target range will receive additional information after t2. The following safety regulations will be followed:

1. Practices with any temperature beyond the target range will receive detailed information on the date and duration of the incident.
2. They will also receive detailed information recommending that the manufacturer’s hotline(s) be contacted for information on how to deal with vaccines exposed to inadequate temperatures, and if additional vaccinations of patients are required to assure patient immunity.
3. In addition, practices will receive the contact data of the Paul Ehrlich Institute, the Federal German agency responsible for vaccines and appropriate management thereof.

### Data collection procedure

#### Practice recruitment

The study will be performed in a random sample of primary care practices in North Rhine-Westphalia, Germany. The sample will be restricted to practices within a 50-km radius of the University Hospital Essen. To support a systematic recruitment process and ensure detailed documentation of all contact attempts and communications with the practices, we will use a response-tracking database. To achieve a high response rate, we will follow a multi-level approach, consisting of up to three invitation letters and up to five phone calls on different days and at different times. The first phone call with each physician is to ensure the eligibility of practice, that is, vaccines are administered, provide additional study information, schedule an appointment for the first practice visit by the study assistant. In order to allow for a nonparticipant analysis, practices refusing participation will be asked to complete a short questionnaire on their practice characteristics (solo/group practice, number of patients quarterly, number of physicians in practice, number of refrigerators used, and availability of a refrigerator thermometer).

#### Randomization

Based on results of the temperature baseline data collection, practices with temperatures outside the target range will be randomized equally to the two study arms (1:1). The randomization procedure is computer-based through www.random.org. No participant will be informed about the study hypotheses in detail.

#### Sample size estimation

The intervention study will be performed in a sample of 46 primary care practices (23 practices per arm) with suboptimal temperature readings at baseline during a 7-day monitoring period (t0). A sample size of 23 practices per group provides an 80 % power to detect anticipated differences between the groups at a significance level of 0.05 %. We assume that 70 % of the practices in the intervention group will reach the temperature target range at follow-up. This assumption is based on two intervention studies of comparable intensity that observed optimal storage conditions in 83 % and 77 % of previously suboptimal practices, using continuous temperature readings as the outcome [18, 19]. Regarding the control group, we expect that 30 % of practices will reach the temperature target range at follow-up due to a study participation effect because of being exposed to temperature monitoring via data logger. This assumption is based on an intervention study that observed optimal temperature readings in 33 % of the control group at follow-up [18].

Owing to the lack of intervention studies available, the power calculation is based on more conservative assumptions.

As the intervention will only be conducted in practices with suboptimal temperatures, a total of 174 practices will have to be recruited for t0. This is based on the assumption that 33.3 % of refrigerators will be outside the target range (n = 58). This percentage is derived from the abovementioned prospective feasibility study in 17 primary care practices with 21 vaccine refrigerators and similar results reported by Bell et al. (2001), Jeremijenko et al. (1996) and Lewis et al. (2001) [14, 18, 20]. Our calculation takes into account a dropout rate of 20 %. This dropout rate is based on our experience and corresponds to reports from other cluster-randomized trials [24]. Using an adaptive design, baseline temperature measurements for t0 will be stopped as soon as the anticipated total sample of n = 58 is reached.

### Statistical analysis

Descriptive statistics will be used to describe baseline characteristics for both study arms with regard to practice characteristics (single/group, number of physicians/medical assistants in practice, number of patients quarterly, average number of vaccinations daily/per week, age structure of patients), participating physician and practice personnel characteristics (sex, age, years in practice/job, part-time/full-time, job content). The effectiveness will be assessed by comparing the intervention and control group and by intragroup comparison (baseline versus follow-up). We will evaluate the effectiveness based on temperature as the main outcome (within range yes/no) by comparing both groups using the chi-square test. For intergroup analyses, all dichotomous secondary outcomes will be evaluated using chi-square tests for each item. In addition, compliance with storage recommendations will be analyzed on the basis of a sum score calculated across all items by using a t-test. For in-group comparisons, McNemar tests will be calculated for dichotomous outcomes and t-tests for dependent samples for sum scores. Temperature monitoring will be measured on an ordinal level to allow for application of the Mann-Whitney U Test (intergroup comparison) and the Wilcoxon signed rank sum test (intragroup comparison). A *P* value <0.05 will be considered significant. All statistical analyses will be performed using IBM SPSS 22® on Windows.

## Discussion

To our knowledge, this will be the first randomized controlled trial evaluating a web-based approach in comparison to a control group addressing the issue of vaccine storage. The topic is important to ensure vaccine effectiveness and safety but currently plays a minor role, at least in German primary care practices. Some of this lack of recognition is due to the fact that this topic is only marginally addressed, if addressed at all, in the medical education of physicians and practice personnel.

Effectively changing medical practice requires a “complex approach focused on different levels, tailored to specific settings and target groups” [21]. To address the issue of vaccine storage, we chose a web-based education that combines a number of benefits. First, while being as effective and satisfactory as traditional educational approaches [25, 26], it addresses both target populations, that is, physicians and practice team members. The tailored presentation of relevant learning content is discussed to increase the appropriateness of the information on a personal level, thereby implying that contents are better remembered and more frequently discussed (for example, [27]). Although computer tailoring has not been studied in healthcare providers, it was proven to be effective in patients’ health education and promotion [28, 29]. Second, an e-learning intervention tool can easily be used for recurrent training, for example, to train new personnel in case of fluctuation or to retrain staff in the form of continuous medical education. Third, in contrast to 1:1 education our web-based learning format can be disseminated on a larger scale at low cost.

Methodologically, the study exhibits a number of strengths. First, in contrast to prior studies, continuous vial temperature using a glycol probe as opposed to single-point measurements of ambient air temperature is used as a primary outcome parameter. Second, to take into account the frequently reported fluctuations in performances, several strategies to intensify the intervention are combined: the intervention is directed at both the individual physician and practice personnel as well as the organizational level. Participants will receive tailored information and a manual for later reference, whereas the practice as an organizational unit will receive managerial support in the form of templates, plans and guides for various aspects of vaccine management, for example, for temperature recording and emergency plans in the event of equipment failure. A manual on vaccine storage management is a novelty to German practices as currently no agreed-upon reference is available, and the official German recommendations for vaccines contain only little information on storage issues. Third, in contrast to some prior interventions lacking in-depth descriptions of intervention contents and implementation processes, we will describe the content of the web-based education intervention in detail to allow for replication and/or building on research findings. Fourth, our web-based intervention is designed for future widespread use in small and larger healthcare institutions at low cost.

## Trial status

Practice recruitment is planned to start in January 2016.

## Abbreviations

AUS: Australia
C: Celsius
CDC: Centers for Disease Control
F: Fahrenheit
UK: United Kingdom
US: United States of America
WHO: World Health Organization
